# Antiallodynic Effect of Herbal Medicine Yokukansan on Peripheral Neuropathy in Rats with Chronic Constriction Injury

**DOI:** 10.1155/2012/953459

**Published:** 2012-02-08

**Authors:** Yasuyuki Suzuki, Hiromasa Mitsuhata, Mitsutoshi Yuzurihara, Yoshio Kase

**Affiliations:** ^1^Tsumura Research Laboratory, Tsumura & Co., 3586 Yoshiwara, Ami-machi, Inashiki-gun, Ibaraki 300-1192, Japan; ^2^Department of Anesthesiology and Pain Medicine, Juntendo Tokyo Koto Geriatric Medical Center, 3-3-20 Shinsuna, Koto-ku, Tokyo 136-0075, Japan

## Abstract

Yokukansan, one of the traditional Japanese herbal medicines, ameliorated neuropathic pain symptoms in patients. In this study, we investigated the effects of yokukansan on neuropathic pain in chronic constriction injury (CCI) model. Oral administration of yokukansan significantly inhibited mechanical and cold allodynia in the von Frey hair or acetone test, respectively. In comparison, amitriptyline, a tricyclic antidepressant, demonstrated moderate, but not significant, antiallodynic effects in the mechanical and cold tests. Yokukansan significantly inhibited the cerebrospinal fluid dialysate level of glutamate that had increased by the stimulation of brush or acetone. Glutamate transporter inhibitors, DL-threo-beta-hydroxy aspartate and dihydrokainate, decreased the yokukansan-induced antiallodynic actions in CCI rats. Our results suggest that yokukansan was confirmed to have antiallodynic effects in CCI rats, which are related to a blockade of glutamatergic neurotransmission via activation of glutamate transporters in the spinal cord.

## 1. Introduction

Chronic pain related to peripheral neuropathy is clinically important. Neuropathic pain is often characterized by pathological symptoms such as hyperalgesia and allodynia, as also reported for spontaneous pain [1, 2]. Mechanical or cold allodynia is a problem that depreciates the quality of life of patients. For the treatment of neuropathic pain, opioid analgesics, tricyclic antidepressants, anticonvulsive agents such as gabapentin, and topical anesthetics have been administered, and nonpharmacological treatments including acupuncture and electrical stimulation have been performed. However, they are not completely effective in ameliorating neuropathic pain, thus new treatments and drugs are necessary [3, 4].

Yokukansan is one of the traditional Japanese medicines called *kampo* in Japan. It is composed of seven kinds of medicinal herbs. Yokukansan has been approved by the Ministry of Health, Labor, and Welfare of Japan as a remedy for neurosis, insomnia, and irritability in children. Recently, yokukansan was reported to improve behavioral and psychological symptoms of dementia (BPSD) including hallucinations, agitation, and aggressiveness in patients with Alzheimer's disease, dementia with Lewy bodies, and other forms of senile dementia [5, 6]. We previously found that yokukansan clinically attenuates neuropathic pain due to postherpetic neuralgia, complex regional pain syndrome, and central cord syndrome and reported that yokukansan ameliorates neuropathic pain symptoms in patients [7].

Animal models of peripheral nerve injury are useful to elucidate the mechanisms underlying neuropathic pain [8] and to improve current clinical treatments [9]. Chronic constriction injury (CCI), an animal model of neuropathic pain proposed by Bennett and Xie [10], produces pronounced behavioral responses and it can reproduce signs noticed in humans, contributing thereby to advance the understanding of neural mechanisms in neuropathic pain disorders [8]. The Bennett and Xie model is commonly used for studies of mononeuropathy characterized by the mechanical and cold allodynia that are produced by chronic constriction of the sciatic nerve. We evaluated the effects of yokukansan on allodynia using rats with CCI-induced neuropathy in this study.

## 2. Methods

### 2.1. Animals

Male Sprague-Dawley (SD) rats weighing 250300 g (Japan SLC Ltd., Hamamatsu, Japan) were used. The animals were allowed free access to water and standard laboratory food (MF, Oriental Yeast, Tokyo, Japan) and kept in a facility at a temperature of 23 ± 3°C and relative humidity of 50 ± 20%, with lights on from 07:00 to 19:00 daily. All experimental procedures were performed according to the “Guidelines for the care and use of laboratory animals” approved by the Laboratory Animal Committee of Tsumura & Co.

### 2.2. Drugs

Yokukansan, which is manufactured by Tsumura & Co. (Tokyo, Japan) and approved for ethical use by the Ministry of Health, Labor, and Welfare of Japan, is a dried extract of the following botanical raw materials: 4.0 parts Atractylodis Lanceae Rhizoma (rhizome of *Atractylodes lancea* De Candolle, Compositae), 4.0 parts Poria (sclerotium of *Poria cocos* Wolf, Polyporaceae), 3.0 parts Cnidii Rhizoma (rhizome of *Cnidium officinale* Makino, Umbelliferae), 3.0 parts Angelicae Radix (root of *Angelica acutiloba* Kitagawa, Umbelliferae), 2.0 parts Bupleuri Radix (root of *Bupleurum falcatum* Linné, Umbelliferae), 1.5 parts Glycyrrhizae Radix (root and stolon of *Glycyrrhiza uralensis* Fisher, Leguminosae), and 3.0 parts Uncaria Uncis Cum Ramulus (hook of *Uncaria rhynchophilla* Miquel, Rubiaceae). Each plant component was identified by its external morphology and authenticated by marker compounds of plant specimens according to the methods of the Japanese Pharmacopoeia and our company's standards. The seven medical herbs were extracted with purified water at 95°C for 1 h, and the extract solution was separated from the insoluble waste and concentrated by removing water under reduced pressure. Spray drying was used to produce a dried extract powder. The yield of the extract was about 15.9%.

Yokukansan and amitriptyline hydrochloride, which was purchased from Wako Chemicals Industries (Osaka, Japan), were dissolved in distilled water and administered by oral gavage. DL-threo-beta-hydroxyaspartate (TBHA, Sigma-Aldrich, St. Louis, MO, USA) was dissolved in phosphate-buffered saline containing 1% dimethyl sulfoxide, and dihydrokainate (DHK, Sigma-Aldrich) was dissolved in PBS.

### 2.3. Chronic Constriction Injury

The chronic constriction injury (CCI) was produced as described previously [10]. Rats were anesthetized with sodium pentobarbital (50 mg/kg, i.p.), and the right common sciatic nerve was exposed at the level of the middle of the thigh by blunt dissection through the biceps femoris muscle. Four ligatures (4–0 monocryl monofilament), about 1 mm apart, were tied loosely around the nerve. The muscle layer and skin layer were closed using silk thread. In sham-operated rats, the right sciatic nerve was exposed but not ligated.

### 2.4. Mechanical Allodynia

The responsiveness to innocuous mechanical stimulation was determined by probing the lateral edge of the plantar hind paw surface with an ascending series of von Frey filaments (1.4–60 g, North Coast Medical, Inc., Morgan Hill, CA, USA). Rats were placed in a metal mesh cage raised 20 cm above the floor, and the animals were allowed to adapt for approximately 20 min or until exploratory behavior ceased. Lifting of the paw due to normal locomotor behavior was ignored. Starting with the smallest force, the von Frey filament was placed on the skin until it bowed slightly, with each filament presented five times at a rate of about 1/s. A different region within the test area was stimulated with each presentation. If a withdrawal response was not elicited, the next ascending von Frey filament was applied until a response occurred. The minimum force-reproducible flexion-withdrawal reflex on five applications of von Frey hairs to the right hind paw was measured as the mechanical withdrawal threshold. The antiallodynic activity was evaluated every 1 h for 4 h after administration of the drugs.

### 2.5. Cold Allodynia

For the assessment of cold allodynia, the rats were placed in a metal mesh cage and allowed to habituate for approximately 20 min or until exploratory behavior ceased. Cold allodynia was measured by squirting 250 *μ*L of acetone onto the midplantar surface of the hind paw as described by Choi et al. [11]. A positive response to acetone was defined as a sharp withdrawal of the hind paw lasting >1 s. Very brief withdrawals lasting <1 s were assigned a value of 0 because they could be occasionally induced by acetone but were never observed after the application of water. The paw elevation time was measured for 1 min with a digital stopwatch from the onset of the paw withdrawal until the paw was rested again on the cage floor for at least 2 s. The antiallodynic activity was evaluated every 1 h for 4 h after administration of the drugs.

### 2.6. Intrathecal Catheters

Intrathecal catheters were implanted in rats according to the method of Yamamoto and Yaksh [12] with minor modifications. Briefly, the atlanto-occipital membrane was incised right below the skull, and a PE-10 polyethylene catheter filled with sterile saline was introduced 9 cm into the spinal cavity so that the catheter tip extended to the rostal edge of the lumbar enlargement. The catheter was externalized on the top of the skull and sealed with a piece of steel wire and the wound closed with 3–0 silk sutures. After a one-week recovery period, animals with normal neurologic function were operated the CCI surgery.

### 2.7. In Vivo Microdialysis

CCI rats under sodium pentobarbital (50 mg/kg, i.p.) anesthesia were implanted with a single dialysis catheter (Marsil Scientific, San Diego, CA) into the subarachnoid space through an incision between the L4–L5 vertebrae. The total length of the catheter was 9 cm, including an active dialysis fiber length of 4 cm (0.2 mm inner diameter, 0.3 mm outer diameter, 11 kDa cut-off). The active site was positioned to span lumbar enlargement segments. The dialysis fiber was connected to the outer lumen of the polyethylene tubing via 1 cm length of fused silica tubing used as stents, with methacrylate adhesive on the outside. Fine gauge wire lengthwise inside the dialysis fiber prevented crimping. The two distal ends of the catheter were tunneled subcutaneously and externalized through the skin in the neck region and plugged with short wire. The experiments were performed at least five days after the implantation of the dialysis catheter. Only rats with normal behavior and no paralysis of the hind limbs after the surgery were included in the microdialysis studies. The rats were used only once for the experiments. After recovery from surgery, microdialysis experiments were carried out with unanesthetized and freely moving animals placed in a Plexiglas chamber (30 cm wide, 40 cm long, 40 cm high). The dialysis catheter was perfused with artificial cerebrospinal fluid (ACSF; 147 mM NaCl, 4.0 mM KCl, 2.3 mM CaCl2, pH 6.4) at a constant flow rate of 2 *μ*L/min. Methylene blue dye was perfused through the dialysis probe to verify the position of the dialysis fiber. The samples were collected as 20 min fractions and frozen at −80°C until analysis. Three consecutive samples were collected for determination of basal levels after an initial washout period of 60 min, and then rats received oral administration of yokukansan (1 g/kg) or distilled water (10 mL/kg). One hour after oral administration, touch stimulation of the affected leg with a brush or cold stimulation with 0.25 mL of acetone was performed every minute for 20 minutes. The glutamate level in the perfusate was determined by a high-performance liquid chromatography system with electrochemical detection with o-phthalaldehyde derivatization. The derivatization was carried out by mixing 30 *μ*L of perfusate and 10 *μ*L of 4 mM o-phthalaldehyde-2-mercaptoethanol at 10°C for 10 min. The chromatographic conditions were column: EICOMPAK SC-5ODS (3.0Φ × 150 mm long, Eicom), mobile phase: 0.1 M phosphate buffer, pH 6.0, containing 29% methanol and 13.4 m M EDTA-2Na, flow rate: 0.5 mL/min, column temperature: 30°C, an applied potential to an Ag/AgCl reference electrode: +600 mV, and working electrode: graphite electrode.

### 2.8. Statistical Analysis

The results are expressed as means ± SEM. Significance was determined using one-way analysis of variance (ANOVA), followed by Dunnett's *t*-test or two-way ANOVA. In all cases, differences of *P* < 0.05 were considered significant.

## 3. Results

**Figure 1:**
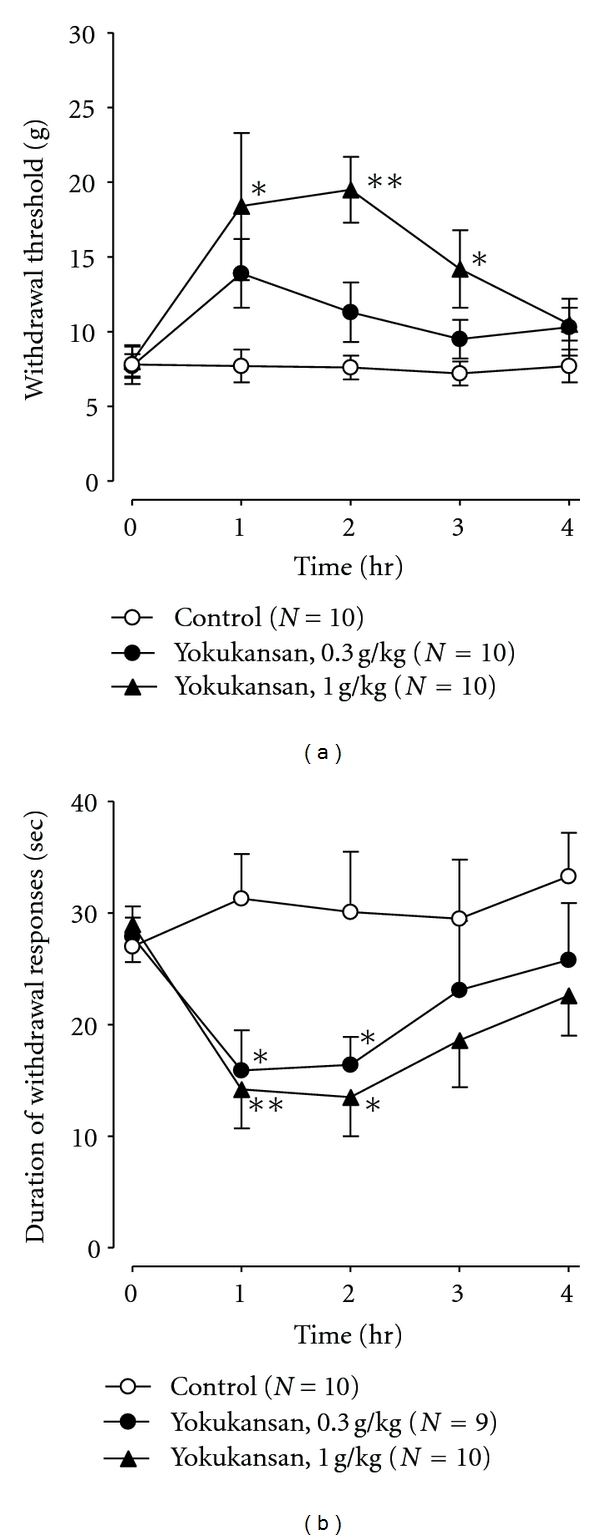
Effects of yokukansan on mechanical (a) and cold (b) allodynia in CCI-induced neuropathic rats. Yokukansan (0.3, 1.0 g/kg, p.o.) or distilled water (DW) was administered orally at time zero. Each point represents the mean ± SEM. *P < 0.05, **P < 0.01 compared with the respective distilled water (DW) treatment group.

**Figure 2:**
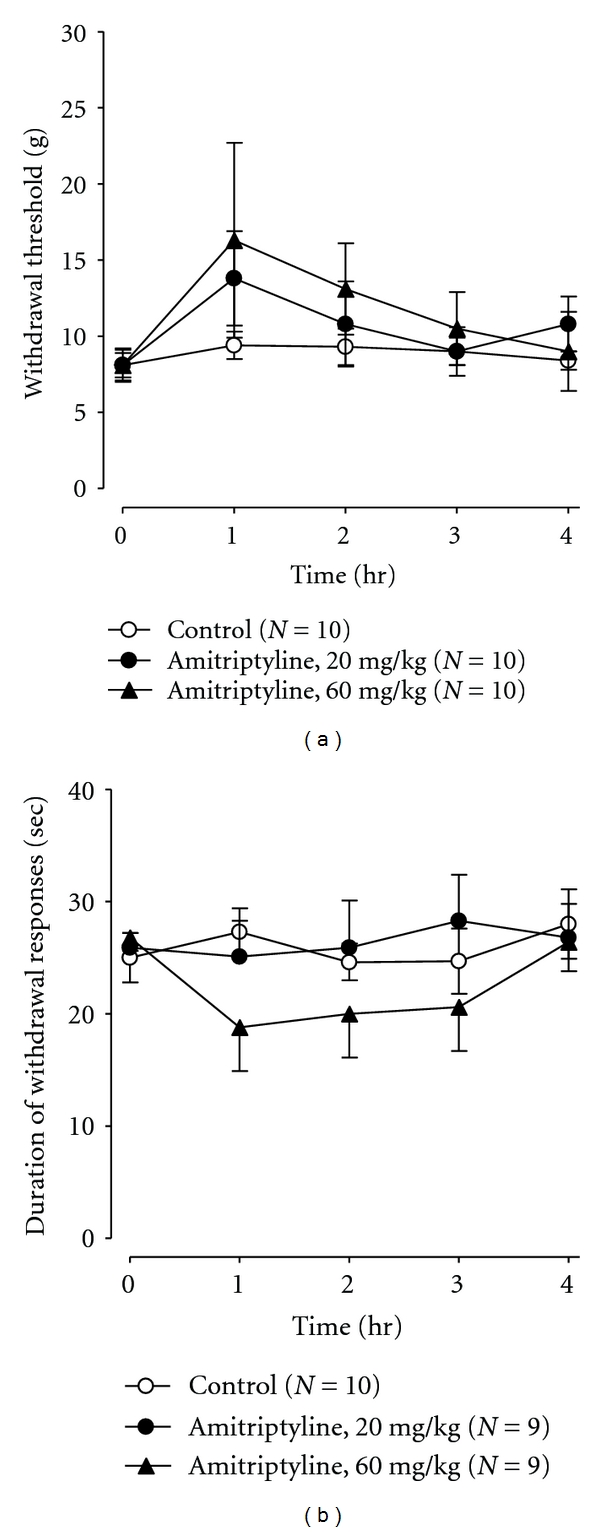
Effects of amitriptyline on mechanical (a) and cold (b) allodynia in CCI-induced neuropathic rats. Amitriptyline (20, 60 mg/kg, p.o.) or distilled water (DW) was administered orally at time zero. Each point represents the mean ± SEM.

In the mechanical allodynia test using von Frey filaments, the withdrawal threshold before drug administration in the CCI group (7.8 g ± 1.3, *N* = 10) was lower than in the Sham group (46.4 g ± 8.3, *N* = 5), suggesting mechanical allodynia (*P* < 0.01, Student's *t*-test). When yokukansan at a dose of 1.0 g/kg was orally administered, mechanical hypersensitivity in response to von Frey hair stimulation was significantly attenuated (Figure 1(a)). In the cold allodynia test with acetone stimulation, the duration of withdrawal responses before drug administration in the CCI group (27.0 ± 2.6 sec, *N* = 10) was longer than in the Sham group (3.3 ± 1.4 sec, *N* = 5), suggesting cold allodynia (*P* < 0.01, Student's *t*-test). Cold allodynia of the injured hind paw was also dose dependently reduced by administration of yokukansan (0.3, 1.0 g/kg, p.o.) when compared with the control (Figure 1(b)). Oral administration of amitriptyline (20, 60 mg/kg) had little effect on mechanical or cold hypersensitivity in response to von Frey hair or acetone drop stimulation of the injured hind paw compared with the controls (Figures 2(a) and 2(b)). These differences were not statistically significant.

**Figure 3:**
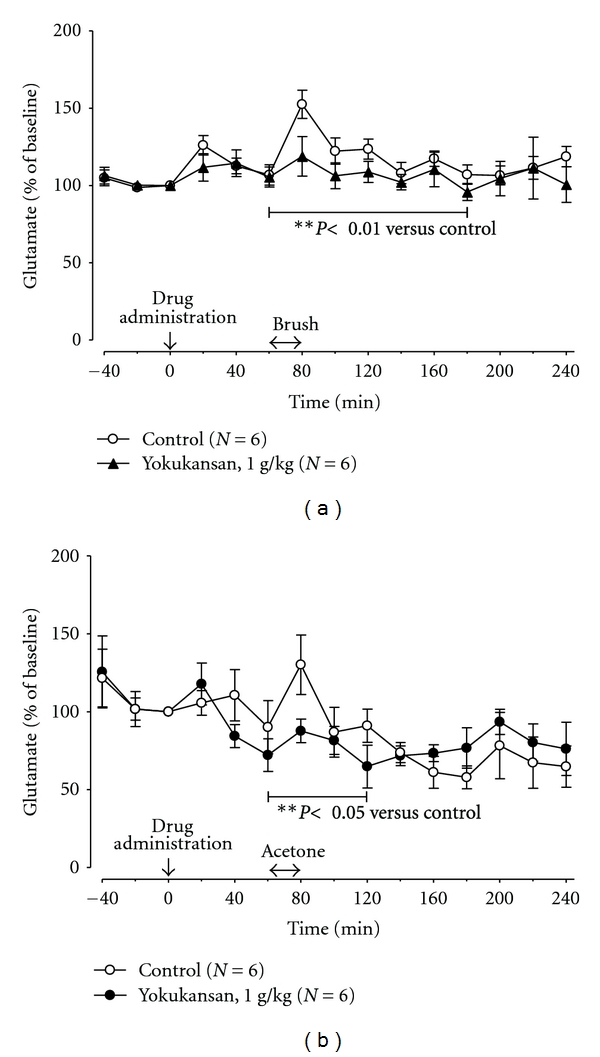
Effect of mechanical (a) or cold (b) stimulation on the levels of lumbar segment enlargement of glutamate in CCI rats and the effects of yokukansan. The time of oral yokukansan (1.0 g/kg) or distilled water (control) administration was regarded as minute 0. Mean ± standard error, *P < 0.05, **P < 0.01 versus control group (two-way ANOVA).

In CCI rats, mechanical stimulation with a brush transiently increased the level of glutamate in the spinal cord measured by microdialysis analysis. Oral administration of yokukansan at 1.0 g/kg inhibited the elevation of glutamate level in the spinal cord between 60 and 180 minutes after administration, during which significant actions were observed in the mechanical allodynia test (*P* < 0.01, two-way variance analysis; Figure 3(a)). Furthermore, cold stimulation with acetone also increased the level of glutamate in spinal cord of CCI rats. Oral administration of yokukansan at 1 g/kg inhibited the elevation of glutamate in the spinal cord between 60 and 120 minutes after administration, during which significant effects were obtained in the cold allodynia test (*P* < 0.05, two-way variance analysis; Figure 3(b)).

**Figure 4:**
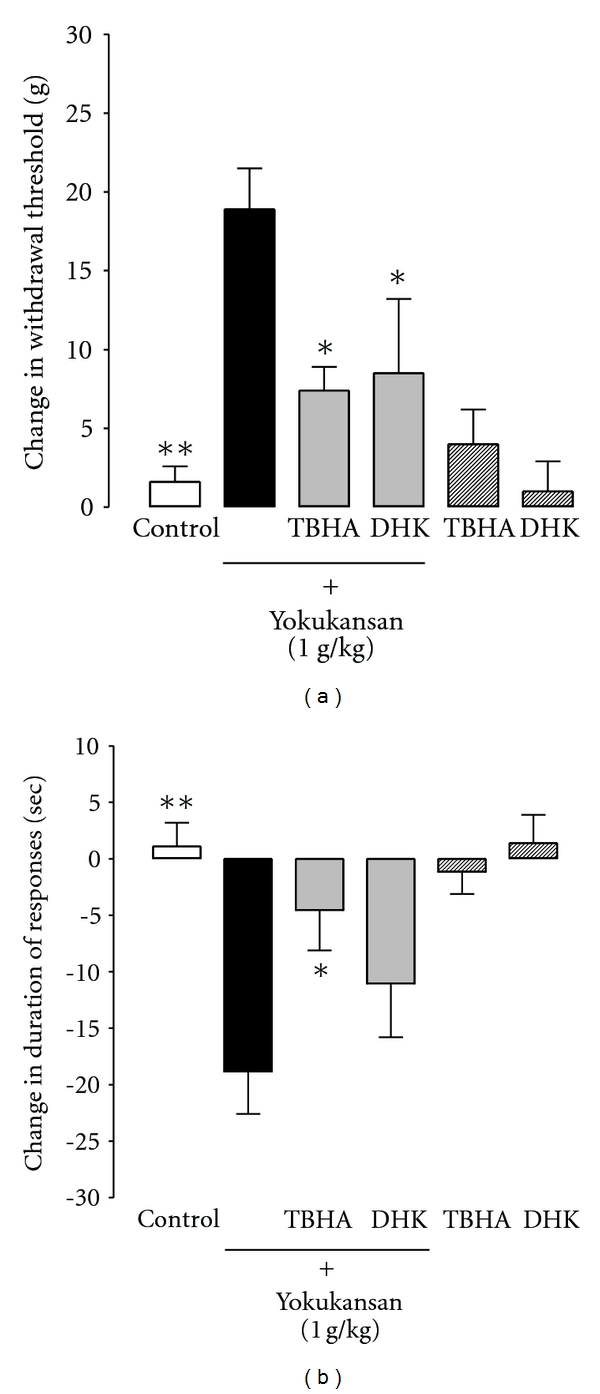
Effect of glutamate transporter inhibitors on the anti-allodynic effects of yokukansan on mechanical (a) or cold (b) stimulation with a von Frey filament or acetone in CCI rats. A glutamate transporter inhibitor, TBHA (1 *μ*g) or DHK (5 mM), was simultaneously intrathecally injected when yokukansan (1.0 g/kg) was orally administered. Differences between values before and 1 hour after yokukansan administration are expressed as the mean ± standard error. *P < 0.05, **P < 0.01 versus yokukansan alone (Dunnett's *t*-test).

In the preliminary microdialysis study, we examined spinal cord glutamate release induced by intrathecal (i.t.) injections of TBHA (1, 3, 10 *μ*g) or DHK (5, 10, 20 mM). As a result, the intrathecal injections of TBHA (1 *μ*g) and DHK (5 mM) did not increase intraspinal glutamate by themselves (data not shown). Therefore we used 1 *μ*g of TBHA and 5 mM of DHK for the behavioral tests. The comparison of measurements on each test one hour after yokukansan administration with the pretreatment values is shown in Figure 4. In the mechanical allodynia test, the intrathecal administration of TBHA (1 *μ*g), which nonspecifically inhibits glutamate transporter-1 (GLT-1) and glutamate-aspartate transporter (GLAST), or DHK (5 mM), a GLT-1-selective inhibitor, significantly reduced the antiallodynia actions of yokukansan at 1 g/kg (Figure 4(a)). In the cold allodynia test, TBHA (1 *μ*g, i.t.) significantly reduced the antiallodynia actions of yokukansan at 1 g/kg. DHK (5 mM, i.t.) inhibited the actions of yokukansan, although there was no significant difference (Figure 4(b)).

## 4. Discussion

Recently we found that yokukansan clinically improves subjective symptoms of neuropathic pain [7]. In the present study, we demonstrated that administration of yokukansan produced antiallodynic effects against mechanical and cold stimuli in the painful peripheral neuropathy rat model with CCI, implying that this antiallodynic effect may be one of the factors that attenuate neuropathic pain in patients.

Tricyclic antidepressants, including amitriptyline, are currently used for neuropathic pain. Amitriptyline is recommended as first-line treatment in painful neuropathy in some clinical guidelines including European Federation of Neurological Societies and National Institute for Health and Clinical Excellence [3, 13]. We used amitriptyline in this experiment to confirm effect of amitriptyline in CCI model rat. Tricyclic antidepressants are considered effective for both steady and lancinating or brief pains, whereas it is more difficult to judge if these drugs also relieve touch-evoked pain [2]. When amitriptyline was administered orally to rats with peripheral nerve injury in this study, mechanical allodynia was only slightly attenuated. This result is consistent with reports that amitriptyline is not effective in mechanical allodynia in CCI models or spinal ligation models [14–16]. Similarly, amitriptyline (60 mg/kg, p.o.) tended to have little effect on cold allodynia, and its effects were not statistically significant in CCI rats. This result is consistent with a previous report by Wang et al. [16]. Collectively, these findings suggest that amitriptyline has limited effect on neuropathic pain. In contrast, yokukansan markedly improved allodynia in the same models.

The results of this study showed that mechanical stimulation with a brush or cold stimulation with acetone increased the glutamate level in the dialysate obtained from the spinal cord of CCI rats. Oral administration of yokukansan at 1 g/kg inhibited such an increase in the dialysate glutamate level related to these stimuli.

The amino acid glutamate is the major excitatory neurotransmitter in the central nervous system [17]. It is well known that the spinal glutamatergic system plays an important role in physiological nociceptive transmission and in the induction of central sensitization, which are associated with neuronal plasticity underlying pathological pain at the spinal level. Glutamate release in the spinal dorsal horn is elicited following peripheral inflammation or nerve injury [18–21]. Indeed, the microdialysis study confirmed an increase in the spinal cord cerebrospinal fluid (CSF) level of glutamate in a rat neuropathic pain model. In a CCI rat neuropathy model, an increase in the spinal dialysate level of glutamate related to the intrathecal injection of substance P was more marked than sham-operated rats [22]. In a rat neuropathic pain model prepared using the method of Seltzer, the glutamate level of dialysate in the dorsal horn of allodynic animals was higher than that of normal animals [23]. These studies suggest that glutamate is closely involved in spinal nociceptive transmission in patients with neuropathic pain and that the spinal CSF level of glutamate is an index of pain. The microdialysis data revealed in this study indicate that a reduction in the spinal CSF level of glutamate is involved in the antiallodynic mechanism of yokukansan in CCI rats.

In recent years, an increase in the extracellular level of glutamate and degeneration of neuronal and astroglial cells were demonstrated in the brains of thiamine-deficient (TD) rats, and yokukansan was found to inhibit not only the TD-induced increase in the extracellular level of glutamate, but also the degeneration of cerebral neurocytes and astrocytes in vulnerable brain regions [24]. These previous findings support the effects of yokukansan on the spinal cord of neuropathic rats obtained in this study. In the present study, the intrathecal injection of TBHA (1 *μ*g), which nonselectively inhibits GLT-1/GLAST, and DHK (5 mM), a selective GLT-1 inhibitor, significantly reduced the effects of yokukansan on mechanical allodynia. TBHA (1 *μ*g, i.t.) significantly inhibited the anticold allodynic effects of yokukansan, while DHK (5 mM) only slightly reduced them. These results suggest that yokukansan exhibits antimechanical allodynic effect via activation of GLT-1, whereas GLAST activation may be related to effect of yokukansan on cold allodynia. It is unclear why the effect of DHK on antiallodynic effect of yokukansan was different between the mechanical and cold tests. These results nonetheless suggest that the antiallodynic effects of yokukansan shown in the present study are related to a blockade of glutamatergic neurotransmission via activation of both glutamate transporters in the spinal cord.

Several studies have suggested that glutamate transporters are involved in the development of hyperalgesia or allodynia associated with neuronal plasticity related to central sensitization following peripheral inflammation and nerve injury. The increase of glutamate reuptake related to the intrathecal administration of a glutamate transporter activator or intraspinal overexpression of glutamate transporters attenuated hyperalgesia and allodynia in rats with inflammatory and neuropathic pain [20, 25]. In contrast, intrathecal injection of glutamate transporter inhibitors into naive animals elevated the spinal extracellular glutamate level and produced spontaneous nociceptive-related behaviors and hyperalgesia [26–28]. On the other hand, CCI in rats significantly reduced spinal glutamate transporter uptake with an associated increase in extracellular glutamate concentration from spinal microdialysates. Furthermore, Sung et al. [29] demonstrated that both the expression and uptake activity of spinal glutamate transporters changed after CCI and contributed to neuropathic pain behaviors in rats. CCI initially upregulated glutamate transporters until at least postoperative day 5, primarily within the ipsilateral spinal cord dorsal horn, which was followed by glutamate transporter downregulation when examined on postoperative days 7 and 14 by Western blotting and immunohistochemistry. Moreover, CCI significantly reduced glutamate uptake activity of spinal glutamate transporters when examined on postoperative day 5, and this reduction was prevented by riluzole (a positive glutamate transporter activity regulator) given intrathecally twice a day on postoperative days 1–4. Riluzole consistently attenuated and gradually reversed neuropathic pain behaviors when a 4-day riluzole treatment was given on postoperative days 1–4 and 5–8, respectively [29]. These results indicate that changes in the expression of spinal glutamate transporters and their glutamate uptake activity may play a critical role in both the induction and maintenance of neuropathic pain after nerve injury via the regulation of regional glutamate homeostasis, a recently elucidated mechanism relevant to the pathogenesis of neuropathic pain.

In comparison, it was demonstrated that yokukansan has ameliorated TD-induced decreases in the glutamate uptake function and the levels of GLAST protein and mRNA. This effect of yokukansan has been linked to its ability to strongly modulate GLAST [30]. Among the seven constituent herbs of yokukansan, *Glycyrrhiza* ameliorated the TD-induced decrease in glutamate uptake by astrocytes. Furthermore, among the eight components of *Glycyrrhiza*, glycyrrhizin and its metabolite 18 beta-glycyrrhetinic acid ameliorated the TD-induced decrease of glutamate uptake in astrocytes. These two substances have also been found to inhibit protein kinase C activity under in vitro conditions [31]. Taken together, these studies provide supporting evidence that the antiallodynic effects of yokukansan, as observed in this study, are associated with the promotion of glutamate reuptake via the activation of glutamate transporters in the spinal cord. Whether the components responsible for such actions of yokukansan are indeed glycyrrhizin and its metabolite, 18 beta-glycyrrhetinic acid, requires further investigation. Recently, Egashira et al. [32] reported that yokukansan enhances the pentobarbital-induced sleep in socially isolated mice and that the GABA_A_-benzodiazepine receptor complex is involved in the effect of yokukansan. Therefore, the participation of GABAergic system in the antiallodynic mechanism of yokukansan also should be examined in the future.

In conclusion, yokukansan showed significant antiallodynic effect in CCI model rats and, therefore, may be useful in the treatment of neuropathic pain. The antiallodynic effects of yokukansan are related to a blockade of glutamatergic neurotransmission via activation of glutamate transporters in the spinal cord.
